# Facet-Selective Growth of Heterostructured Molecular Crystals Enabled by a Solid-Solution-Mediated Strategy

**DOI:** 10.1021/jacsau.6c00053

**Published:** 2026-03-20

**Authors:** Tomoya Fukui, Masahiro Tsuchiya, Naoya Mita, Takanori Fukushima

**Affiliations:** † Laboratory for Chemistry and Life Science, Institute of Integrated Research, 163706 Institute of Science Tokyo, 4259 Nagatsuta, Midori-ku, Yokohama 226-8501, Japan; ‡ Department of Chemical Science and Engineering, 163706 Institute of Science Tokyo, 4259 Nagatsuta, Midori-ku, Yokohama 226-8501, Japan; § Research Center for Autonomous Systems Materialogy (ASMat), Institute of Integrated Research, 163706 Institute of Science Tokyo, 4259 Nagatsuta, Midori-ku, Yokohama 226-8501, Japan

**Keywords:** molecular crystals, solid solutions, on-seed-surface crystallization, heterostructures, lattice mismatch, facet-selective growth

## Abstract

Heterojunction interfaces formed between different crystalline materials often provide platforms for emergent functions; however, their construction in molecular crystals remains highly challenging. This difficulty arises because even slight mismatches in lattice parameters or packing geometry can destabilize interfaces, drastically narrowing the tolerance for heteroepitaxy between different molecular crystals. Here, we present a solid-solution-mediated approach that enables on-seed-surface crystallization to form heterostructured molecular crystals from combinations of metal complexes that do not form heterojunctions. Using Fe^II^ and Co^II^ complexes bearing a common tridentate ligand, we show that on-seed-surface crystallization fails because of substantial interfacial lattice mismatch. In contrast, solid-solution crystals composed of Fe^II^ and Co^II^ complexes can serve as secondary components that establish lattice matching with the Fe^II^ seed crystal, leading to the formation of core–shell crystals. Notably, systematic variation of the Fe^II^/Co^II^ feed ratio in the solid-solution composition reveals that the lattice mismatch at the heterojunction becomes increasingly anisotropic as the Fe^II^ content decreases, depending on the crystallographic direction. Under appropriate conditions, this anisotropy gives rise to facet-selective on-seed-surface crystallization of the solid-solution component from the Fe^II^ seed, resulting in dumbbell-like crystals in which secondary segments grow from specific facets of the seed crystal. Single-crystal X-ray analysis further shows that the secondary segments adopt the crystal structure of the seed despite their very low Fe^II^ content. This demonstrates the utility of solid-solution-mediated lattice matching as a strategy for constructing heterostructured molecular crystals.

## Graphical abstract

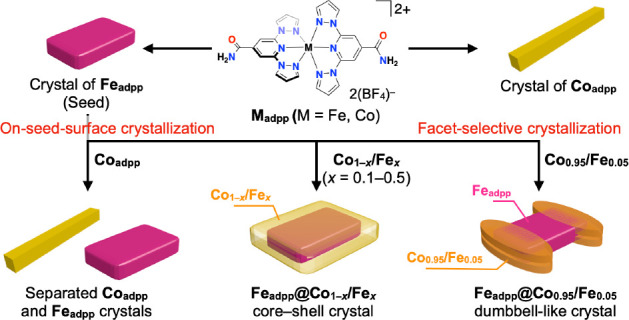

## Introduction

Heterojunction interfaces formed between different crystalline materials often provide platforms for emergent functions. For constructing such heterojunctions, facet-selective crystal growth, in which a secondary component nucleates on specific crystallographic faces, is a promising approach^1–4^ as demonstrated in various materials, including inorganic nanocrystals,^1–9^ metal–organic frameworks (MOFs),^10–13^ and crystalline polymer micelles.^14–17^ However, achieving facet-selective crystallization in molecular crystals is particularly challenging. Unlike inorganic solids, where atoms are continuously connected by strong chemical bonds, molecular crystals are constructed from discrete molecules held together only by weak and highly directional intermolecular interactions. Hence, even slight mismatches in lattice parameters or packing geometry can destabilize the interface, drastically narrowing the tolerance for heteroepitaxy between different components. Consequently, successful examples have been limited to microcrystalline assemblies consisting of π-conjugated molecules.^18–22^

We have recently reported that crystals of a spin-crossover Fe^II^ complex (**Fe**_**cdpp**_) can serve as seeds for the on-seed-surface growth of nonspin-crossover complexes (**Co**_**cdpp**_ and **Zn**_**cdpp**_), giving core–shell crystals in which the shell uniformly wraps around the entire surface of the **Fe**_**cdpp**_ seed (Figure 1a).^23^ All of these complexes consist of the same tridentate ligand, 4-carboxy-2,6-di­(pyrazol-1-yl)­pyridine (cdpp).^23–25^ Notably, the crystal structures of the **Co**_**cdpp**_ and **Zn**_**cdpp**_ shells differ from those obtained by their spontaneous crystallization, instead inheriting the crystal structure of the **Fe**_**cdpp**_ core. Moreover, the spin-transition temperature of the core crystal varies with the degree of local interfacial lattice mismatch at the heterojunction. Despite these insights, a fundamental question remained unresolved: what governs the directionality of on-seed-surface growth, namely, which crystallographic faces are selected for shell nucleation? This motivated us to examine how subtle changes in molecular structure influence the outcome of on-seed-surface crystallization.

**Figure 1:**
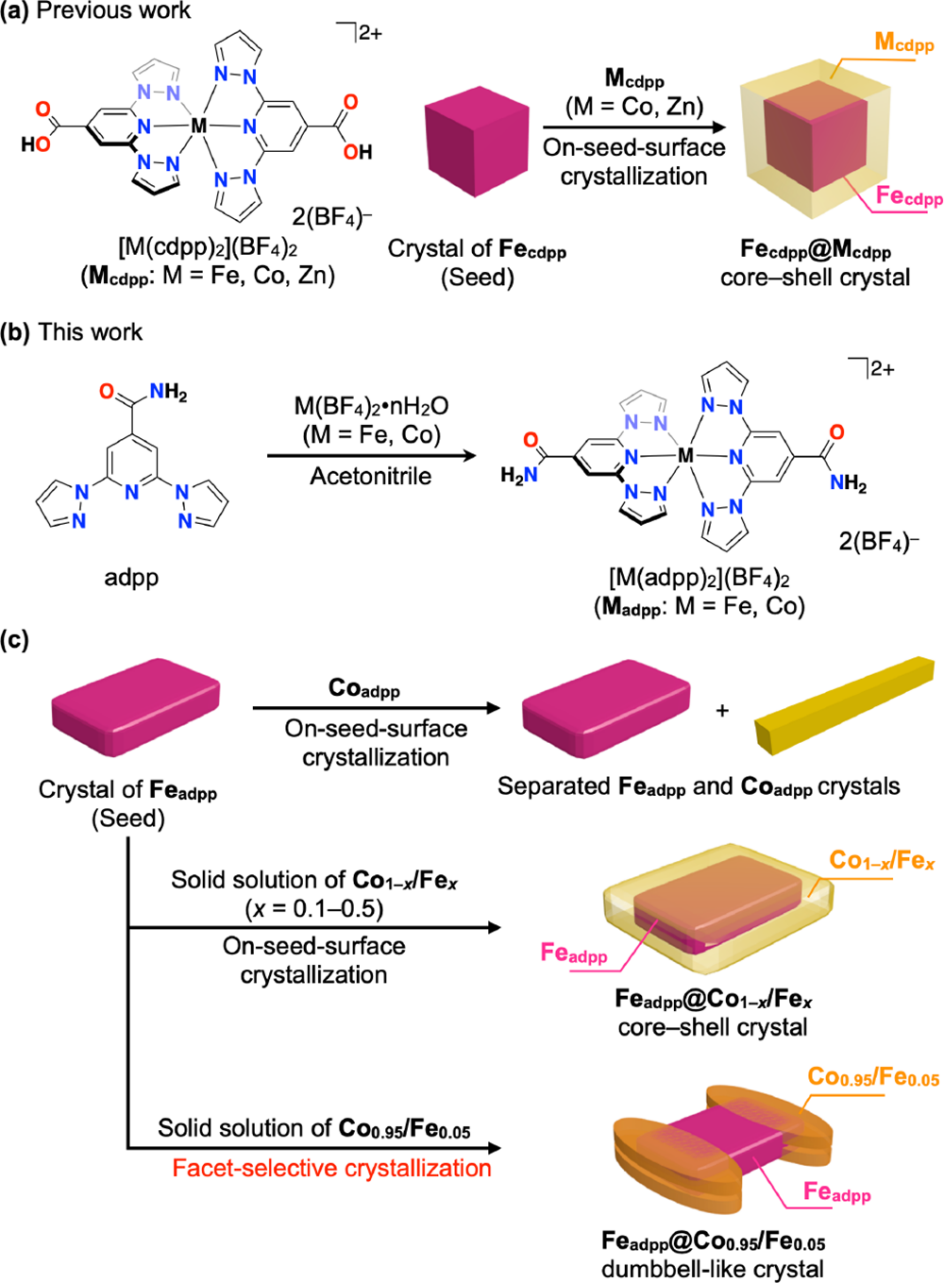
Schematic illustration of (a) the formation of previously reported core–shell crystals of **M**_**cdpp**_ [M = Fe, Co, Zn; cdpp: 4-carboxy-2,6-di­(pyrazol-1-yl)­pyridine] and (b) the preparation of metal complexes **M**_**adpp**_ (M = Fe, Co) using 2,6-bis­(pyrazol-1-yl)­pyridine-4-carboxamide (adpp) as the ligand. (c) Summary of crystallization outcomes obtained via on-seed-surface growth in the present study.

In this study, we used Fe^II^ and Co^II^ complexes bearing the tridentate ligand 2,6-bis­(pyrazol-1-yl)­pyridine-4-carboxamide^26^ (adpp) (Figure 1b). Initial experiments revealed that pure [Co­(adpp)_2_]­(BF_4_)_2_ (hereafter denoted as **Co**_**adpp**_) crystals do not grow on [Fe­(adpp)_2_]­(BF_4_)_2_ (**Fe**_**adpp**_) seeds and instead crystallize independently due to significant lattice mismatch between the two crystals (Figure 1c). To deal with this mismatch, we adopted the solid-solution-mediated strategy, which is commonly used in inorganic materials^27–29^ to systematically adjust the lattice parameters. When **Co**_**adpp**_ was replaced with solid solutions of [Co_1–*x*_Fe_*x*_(adpp)_2_]­(BF_4_)_2_ (**Co_1–*x*_/Fe_*x*_**, *x* denotes the feed ratio of Fe to Co), seeded growth on **Fe**_**adpp**_ became possible, yielding core–shell crystals composed of an **Fe**_**adpp**_ core and a **Co_1–*x*_/Fe**_***x***_ shell (Figure 1c). Here we show that varying the Fe content in the solid solution adjusts the lattice parameters of the shell, which in turn modulates the degree of lattice mismatch at the heterojunction. Remarkably, at low Fe content (ca. 5 mol % in the feed), on-seed-surface growth occurs selectively on specific facets of the **Fe**_**adpp**_ crystals, resulting in the formation of dumbbell-like heterostructures (Figure 1c). These results demonstrate that facet-selective on-seed-surface crystallization in molecular crystals can be achieved by solid–solution-mediated lattice matching, thus bridging a long-standing gap between inorganic and molecular heterostructure design.

## Results and Discussion

### Attempted On-Seed-Surface Crystallization of Co_adpp_ from a Fe_adpp_ Crystal

2,6-Bis­(pyrazol-1-yl)­pyridine-4-carboxamide (adpp) was synthesized according to a previously reported procedure.^26^ Single crystals of **Fe**_**adpp**_ were prepared using a liquid–liquid diffusion method. Thus, an acetonitrile solution of a mixture of adpp (1.0 equiv) and Fe­(BF_4_)_2_·6H_2_O (0.5 equiv) was layered over a concentrated acetonitrile solution of tetrabutylammonium tetrafluoroborate (TBABF_4_, 6.6 M). The two-phase mixture was allowed to stand at 25 °C for 2 days, giving single crystals of **Fe**_**adpp**_ (Figure 2a). **Co**_**adpp**_ crystals were obtained by vapor diffusion of diethyl ether into an acetonitrile solution of adpp (1.0 equiv) and Co­(BF_4_)_2_·6H_2_O (0.5 equiv) (Figure 2b).

**Figure 2:**
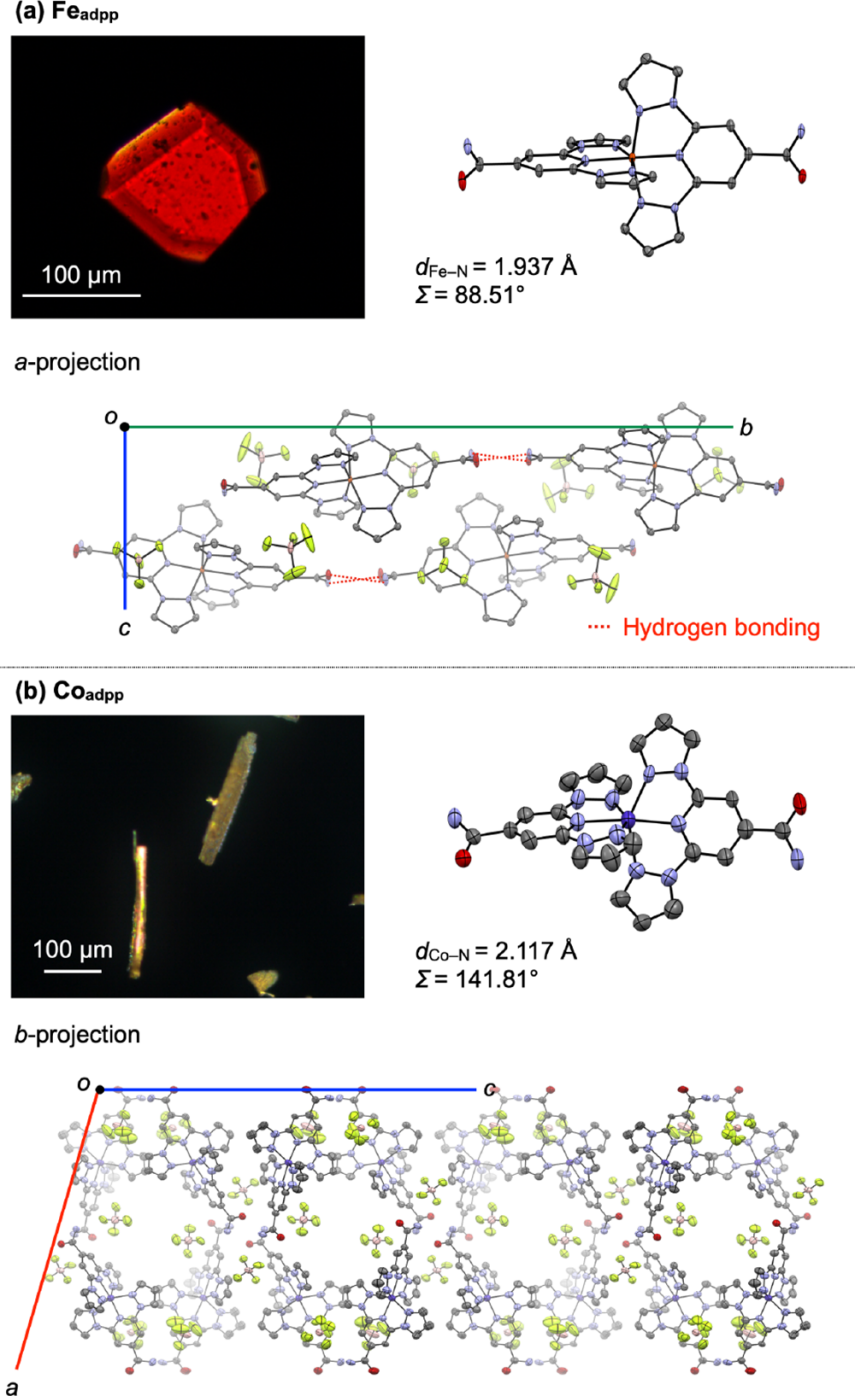
POM images and X-ray crystal structures (molecular and packing views) of (a) **Fe**_**adpp**_ and (b) **Co**_**adpp**_. Color code: carbon = gray, nitrogen = blue, oxygen = red, iron = orange, cobalt = purple, fluorine = green, boron = pink. Hydrogen atoms are omitted for clarity. For panel (b), contributions of solvent molecules have been removed using the SQUEEZE program.^30^

Figure 2 shows the crystal structures of **Fe**_**adpp**_ and **Co**_**adpp**_ at 93 K, together with polarized optical microscopy (POM) images of the corresponding single crystals. The average bond length (*d*_M–N_, M = Fe, Co) and the octahedral distortion parameter (Σ)^31,32^ for **Fe**_**adpp**_ are 1.937 Å and 88.51°, respectively (Table S1). **Fe**_**adpp**_ crystallizes in a monoclinic system with space group *P*2_1_/*n* and forms a one-dimensional hydrogen-bonded chain along the *b*-axis (Figure 2a). On the other hand, **Co**_**adpp**_ cocrystallizes with solvent molecules, leading to a packing arrangement that differs from the one observed for **Fe**_**adpp**_ (Figure 2b). The asymmetric unit contains two independent **Co**_**adpp**_ molecules, whose *d*_Co–N_ and Σ values are 2.117 Å/141.81° and 2.107 Å/131.76°, respectively (Table S1). These values are significantly larger than those of **Fe**_**adpp**_, indicating a higher degree of distortion in the coordination geometry.

We first examined on-seed-surface crystal growth of **Co**_**adpp**_ from **Fe**_**adpp**_ crystals (Figure 1c). Thus, we immersed **Fe**_**adpp**_ crystals in an acetonitrile solution containing adpp (1.0 equiv) and Co­(BF_4_)_2_·6H_2_O (0.5 equiv), into which a diethyl ether vapor was allowed to diffuse at 25 °C (Figure 3a). After 8 h, crystallization of **Co**_**adpp**_ was observed (Figure S1), while no crystal growth occurred on the surface of the seed crystals, even after prolonged incubation. Consequently, the two components crystallized independently (Figure 3b).

**Figure 3:**
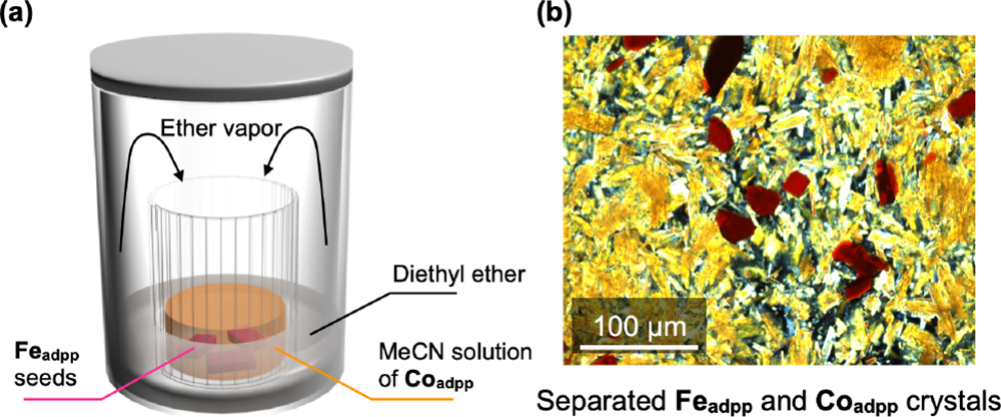
(a) Schematic illustration of the experimental setup for on-seed-surface crystallization of **Co**_**adpp**_ using **Fe**_**adpp**_ seeds. (b) POM image showing separated **Fe**_**adpp**_ (red) and **Co**_**adpp**_ (yellow) crystals obtained after attempted on-seed-surface crystallization.

To understand why on-seed-surface crystallization fails despite the structural similarity of the adpp ligands, we compared the present system to our previously reported **Fe**_**cdpp**_-**Co**_**cdpp**_ one.^23^ The replacement of the carboxylic acid group in cdpp with a carboxamide group in adpp alters the hydrogen-bonding network and packing motif, resulting in crystallographic incompatibility and pronounced lattice mismatch. In **Fe**_**cdpp**_-**Co**_**cdpp**_, **Co**_**cdpp**_ inherits the packing arrangement of the **Fe**_**cdpp**_ seed, enabling the formation of core–shell crystals. In contrast, **Co**_**adpp**_ cannot adapt to the packing structure of **Fe**_**adpp**_ and therefore cannot undergo on-seed-surface crystallization from **Fe**_**adpp**_. The much larger Co–N bond lengths and distortion parameters Σ of **Co**_**adpp**_ introduce substantial interfacial lattice mismatch relative to **Fe**_**adpp**_ (Table S1), likely preventing seeded growth. We assume that even modest differences in coordination geometry can strongly influence the feasibility of on-seed-surface growth in molecular crystals.

### Preparation and Characterization of Solid-Solution Crystals

To achieve on-seed-surface crystallization even in systems with a large degree of lattice mismatch, we examined a solid-solution-mediated approach, which is based on Vegard’s law,^33,34^ well-established in inorganic systems.^27–29^ Solid-solution crystals of [Co_1–*x*_Fe_*x*_(adpp)_2_]­(BF_4_)_2_ (**Co_1–*x*_/Fe**_***x***_, *x* denotes the feed ratio of Fe to Co) were prepared from acetonitrile containing adpp by varying the feed ratio of Fe­(BF_4_)_2_·6H_2_O to Co­(BF_4_)_2_·6H_2_O from 1:9 to 5:5, and allowing diethyl ether vapor to diffuse slowly into the system. The plate-like crystals of **Co**_**1–*x***_ **/Fe**_***x***_ obtained from lower Fe feed ratios ranging from 1:9 to 4:6 are fragile, whereas those obtained from the 5:5 mixture are robust and well-defined in shape (Figure S2). To precisely determine the Fe/Co composition of the **Co_1–*x*_/Fe**_***x***_ crystals, we performed scanning electron microscopy with energy-dispersive X-ray (SEM-EDX) spectroscopy and X-ray fluorescence (XRF) spectroscopy (Figures S3 and S4). Both measurements revealed a nonlinear relationship between the Fe content and feed ratio, particularly at higher feed ratios. Accordingly, the Fe/Co composition in the resulting solid-solution crystals deviates from the feed ratio of Fe­(BF_4_)_2_·6H_2_O and Co­(BF_4_)_2_·6H_2_O used in their preparation.

The structures of the **Co**_**1–** ***x***_ **/Fe**_***x***_ solid-solution crystals, obtained from Fe:Co feed ratios ranging from 1:9 to 4:6, were evaluated using powder X-ray diffraction (PXRD) at 93 K (Figure 4a), and showed good agreement with that of pure **Co**_**adpp**_. Notably, however, the PXRD pattern of the crystal obtained from a 5:5 feed ratio is virtually identical to that of **Fe**_**adpp**_. Single-crystal X-ray diffraction analysis further confirmed that **Co**_**0.5**_ **/Fe**_**0.5**_ crystallizes in a monoclinic system with *P*2_1_/*n* space group (Figure 4b), which is the same as **Fe**_**adpp**_. The average bond length (*d*_Fe/Co–N_) and Σ were determined to be 2.002 Å and 107.4°, respectively (Table S1). These values are closer to those observed for **Fe**_**adpp**_, rather than **Co**_**adpp**_, suggesting that the **Co**_**0.5**_ **/Fe**_**0.5**_ solid-solution adopts a **Fe**_**adpp**_-like structure.

**Figure 4:**
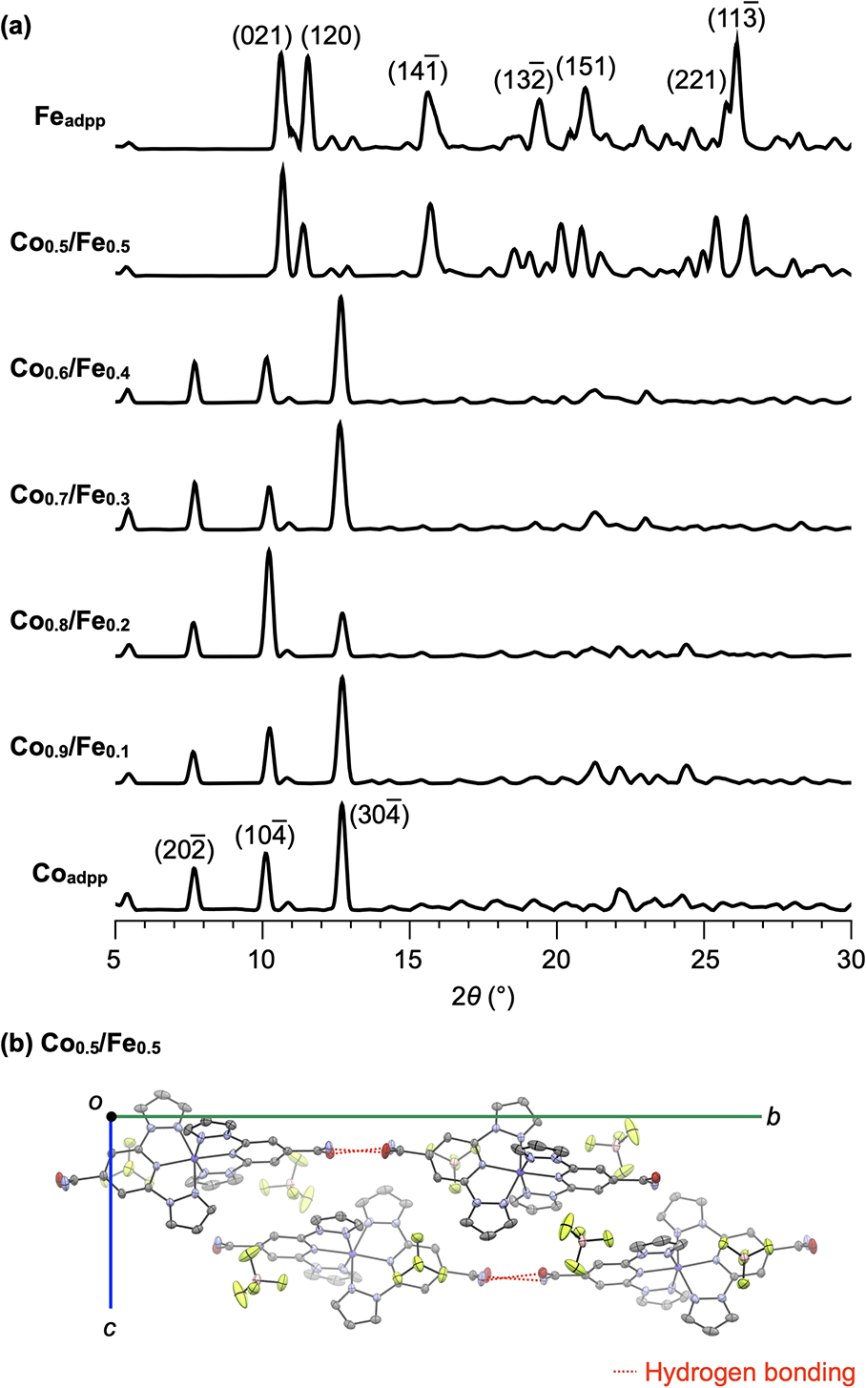
(a) PXRD patterns of bulk samples of **Fe**_**adpp**_, **Co**_**1–** ***x***_ **/Fe**_***x***_ (*x* = 0.1, 0.2, 0.3, 0.4, 0.5), and **Co**_**adpp**_, measured for crystals coated with immersion oil at 93 K. (b) Crystal structure of **Co**_**0.5**_/**Fe**_**0.5**_ viewed along the *a*-axis, with atomic displacement ellipsoids drawn at the 50% probability level. Color code: carbon = gray, nitrogen = blue, oxygen = red, cobalt/iron = purple, fluorine = green, boron = pink. Hydrogen atoms are omitted for clarity (see also Figure S5).

### On-Seed-Surface Crystallization of Solid–Solution Co_1–*x*_/Fe_*x*_ from Fe_adpp_

We found that, unlike in the case of the pure **Co**_**adpp**_-**Fe**_**adpp**_ system, selective crystal growth of solid-solution **Co**_**1–** ***x***_ **/Fe**_***x***_ crystals from **Fe**_**adpp**_ seeds can be achieved. For example, **Fe**_**adpp**_ crystals were added to an acetonitrile solution containing adpp (1.0 equiv), Co­(BF_4_)_2_·6H_2_O (0.25 equiv), and Fe­(BF_4_)_2_·6H_2_O (0.25 equiv), and then diethyl ether vapor was allowed to diffuse into this mixture at 25 °C (Figure S6). After 1 day at 25 °C, the formation of rhombus-shaped core–shell crystals was observed (Figure 5a, top). The SEM-EDX image of the resulting crystals (hereafter denoted as **Fe**_**adpp**_ **@Co**_**0.5**_ **/Fe**_**0.5**_) clearly shows a heterojunction interface between a **Fe**_**adpp**_ core and a **Co**_**0.5**_ **/Fe**_**0.5**_ shell (Figure 5a, bottom). SEM-EDX elemental analysis showed that the shell segment has an Fe/Co atomic composition ratio of 48.8:51.2, reflecting the metal feed ratio used in their on-seed-surface crystallization (Table S2).

**Figure 5:**
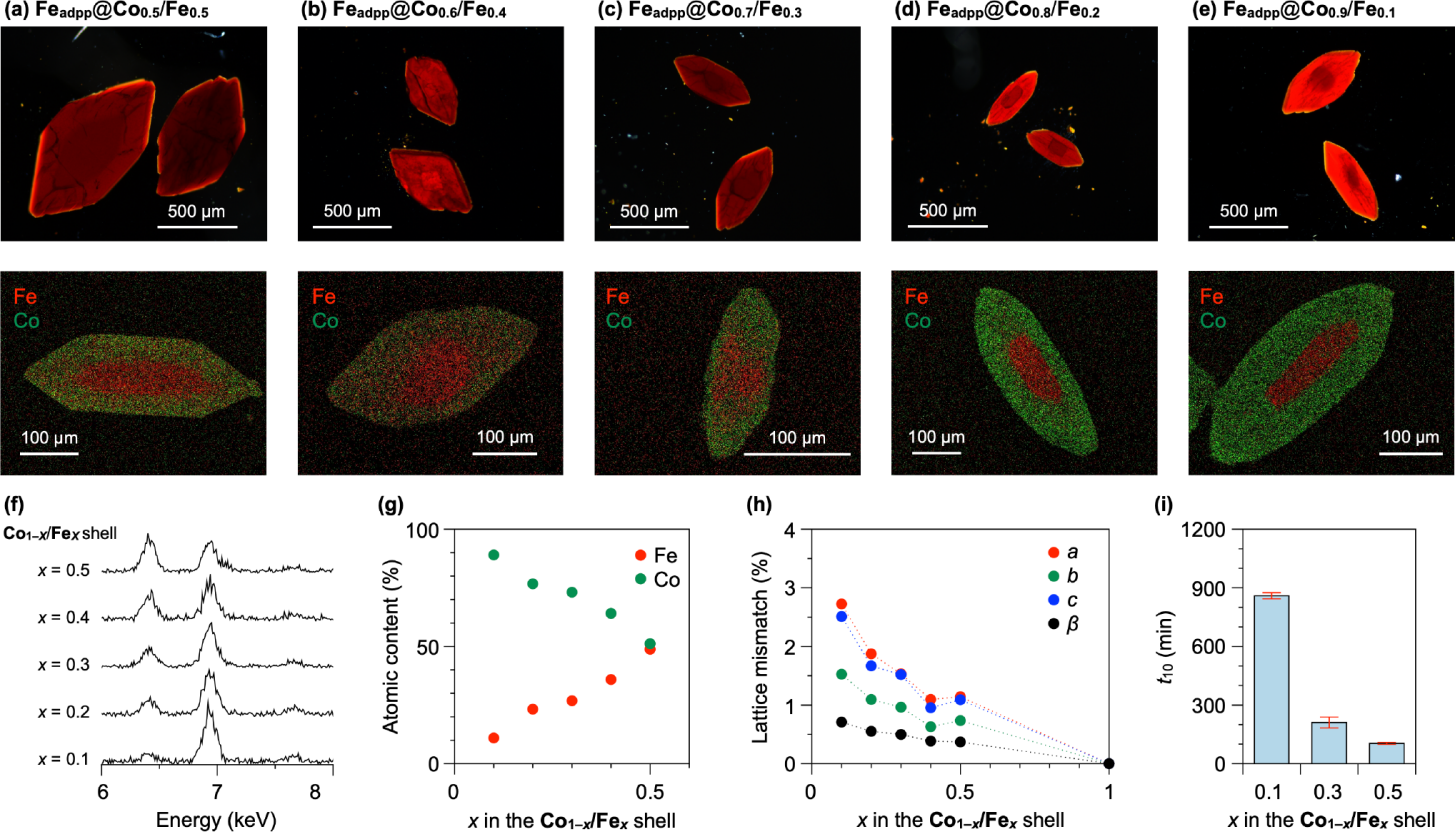
POM (top) and SEM-EDX elemental mapping (bottom) images of core–shell crystals of (a) **Fe**_**adpp**_ **@Co**_**0.5**_ **/Fe**_**0.5**_, (b) **Fe**_**adpp**_ **@Co**_**0.6**_ **/Fe**_**0.4**_, (c) **Fe**_**adpp**_ **@Co**_**0.7**_ **/Fe**_**0.3**_, (d) **Fe**_**adpp**_ **@Co**_**0.8**_ **/Fe**_**0.2**_, and (e) **Fe**_**adpp**_ **@Co**_**0.9**_ **/Fe**_**0.1**_. (f) EDX spectra of the shell segments of **Fe**_**adpp**_ **@Co**_**1–** ***x***_ **/Fe**_***x***_ (*x* = 0.1–0.5). (g) Elemental compositions of Fe and Co in the shell, determined by SEM-EDX analysis, plotted against the Fe­(BF_4_)_2_·6H_2_O/Co­(BF_4_)_2_·6H_2_O feed ratio. (h) Lattice mismatches in the *a* (red), *b* (green), *c* (blue), and β (black) parameters between the **Fe**_**adpp**_ core and the **Co**_**1–** ***x***_ **/Fe**_***x***_ shell, plotted against Fe content *x* in the shell. The lattice mismatches were calculated as percentage differences, as defined in Table S3 (see the Supporting Information). (i) Induction period (*t*_10_) of shell growth, determined by real-time optical microscopy monitoring, plotted against Fe content *x* in the shell (see also Figure S8).

A shell segment was carefully removed from **Fe**_**adpp**_ **@Co**_**0.5**_ **/Fe**_**0.5**_ and subjected to single-crystal X-ray analysis at 93 K, revealing a monoclinic system with space group *P*2_1_/*n*, identical to that of **Fe**_**adpp**_ (Figure S7). The *d*_Fe/Co–N_ and Σ values in the **Co**_**0.5**_ **/Fe**_**0.5**_ shell were 1.987 Å and 102.86°, respectively, both larger than those of pure **Fe**_**adpp**_ (Figure S7). The relative differences in the lattice constants *a*, *b*, *c*, and β between the shell and the core are 1.14, 0.73, 1.09, and 0.37%, respectively (Table S3).

On-seed-surface crystallization of **Co**_**1–** ***x***_ **/Fe**_***x***_ (*x* = 0.1–0.4) through variation of the metal feed ratios under otherwise identical conditions successfully gave **Fe**_**adpp**_ **@Co**_**1–** ***x***_ **/Fe**_***x***_ core–shell crystals (*x* = 0.1–0.4) featuring well-defined heterojunctions (Figure 5b–e, top). SEM-EDX analysis confirmed the presence of both Fe and Co in the shell segments without any detectable local compositional segregation at the spatial resolution of the measurements (Figure 5b–e, bottom). Importantly, the Fe/Co atomic composition ratios were nearly identical to the corresponding metal feed ratios (Figure 5f,g and Table S2). Notably, as also revealed by single-crystal analysis, each shell segment in **Fe**_**adpp**_ **@Co**_**1–** ***x***_ **/Fe**_***x***_ adopts the same crystal structure as **Fe**_**adpp**_ regardless of its Fe content (Figure S7), while the lattice mismatch between the core and shell increases systematically upon decreasing Fe content (Figure 5h and Table S3).

Noteworthy is that while spontaneous crystallization of **Co**_**1–** ***x***_ **/Fe**_***x***_ (*x* = 0.1–0.4) solid solutions results in **Co**_**adpp**_-type structures (Figures 2b and 4a), their on-seed-surface crystallization from **Fe**_**adpp**_ leads to **Fe**_**adpp**_-type structures (Figure S7). Obviously, the structure of **Co**_**1–** ***x***_ **/Fe**_***x***_, in the shell segments of the core–shell **Fe**_**adpp**_ **@Co**_**1–** ***x***_ **/Fe**_***x***_ crystals, is directed by the symmetry and lattice parameters of the seed crystal. Such seed-induced structural control contrasts sharply with intrinsic crystallization pathways and presents a promising approach to realize molecular architectures that are otherwise inaccessible through spontaneous crystal growth.

Real-time optical microscopy monitoring allowed visualization of the time-course growth of the core–shell crystals. As shown in Figure S8, the onset of shell growth became slower as the Fe content decreased. More quantitatively, based on time-resolved analysis, the induction period (*t*_10_), defined as the time required for the shell area to reach 10% of its final value, increased with decreasing Fe content (Figure 5i). Therefore, it is reasonable to consider that larger lattice mismatches prevent secondary nucleation events at the seed–solution interface, slowing shell formation.

In conventional seeded growth or template-directed crystallization strategies, heterostructure formation generally relies on intrinsic lattice compatibility between two discrete crystals, and the crystallographic orientation of the secondary component is inherited from the seed surface. In contrast, the solid–solution-mediated strategy employed here enables lattice matching to be tuned continuously through compositional control of the secondary component, thereby reducing lattice mismatch and allowing heterostructure formation even between two discrete crystals that do not exhibit intrinsic lattice compatibility.

### Facet-Selective Growth of Heterostructured Crystals

Notably, the lattice mismatch between the **Fe**_**adpp**_ core and the **Co**_**1–** ***x***_ **/Fe**_***x***_ shell varies anisotropically with the Fe content (Figure 5h and Table S3). The deviations in the *a*- and *c*-axes increase substantially as the Fe content decreases, whereas the changes in the *b*-axis and β-angle remain relatively small. Importantly, the intermolecular hydrogen-bonding motif remains unchanged across the solid-solution series, whereas the local coordination geometry (e.g., average metal–ligand bond lengths and Σ values) varies systematically with composition (Figure S7). Since the coordination octahedra are oriented along specific crystallographic directions within the lattice, these composition-dependent geometric changes are transmitted through the packing in a direction-dependent manner, leading to larger lattice mismatches along the *a* and *c* directions than along *b*. This pronounced anisotropy led us to hypothesize that, at sufficiently low Fe content, on-seed-surface growth is inhibited on facets with large lattice mismatches, while it remains feasible on facets with more compatible lattice parameters.

To test this hypothesis, we performed on-seed-surface crystallization using a precursor solution containing Co­(BF_4_)_2_·6H_2_O and Fe­(BF_4_)_2_·6H_2_O in a 95:5 molar ratio, thereby introducing a larger anisotropic lattice mismatch than in **Fe**_**adpp**_ **@Co**_**0.9**_ **/Fe**_**0.1**_. Remarkably, under these conditions, dumbbell-like crystals of **Fe**_**adpp**_ **@Co**_**0.95**_ **/Fe**_**0.05**_ formed (Figure 6a,b). Real-time optical microscopy showed that the secondary segments, which were confirmed by SEM-EDX to contain Fe (Figure 6c), nucleated and grew exclusively from specific facets of the **Fe**_**adpp**_ seed (Figure S9). Single-crystal X-ray analysis of a truncated secondary segment revealed that, despite its minimal Fe content, it adopts the same crystal structure as the **Fe**_**adpp**_ seed (Figure S10), indicating that the on-seed-surface crystallization proceeds in a topotactic manner.^23^

**Figure 6:**
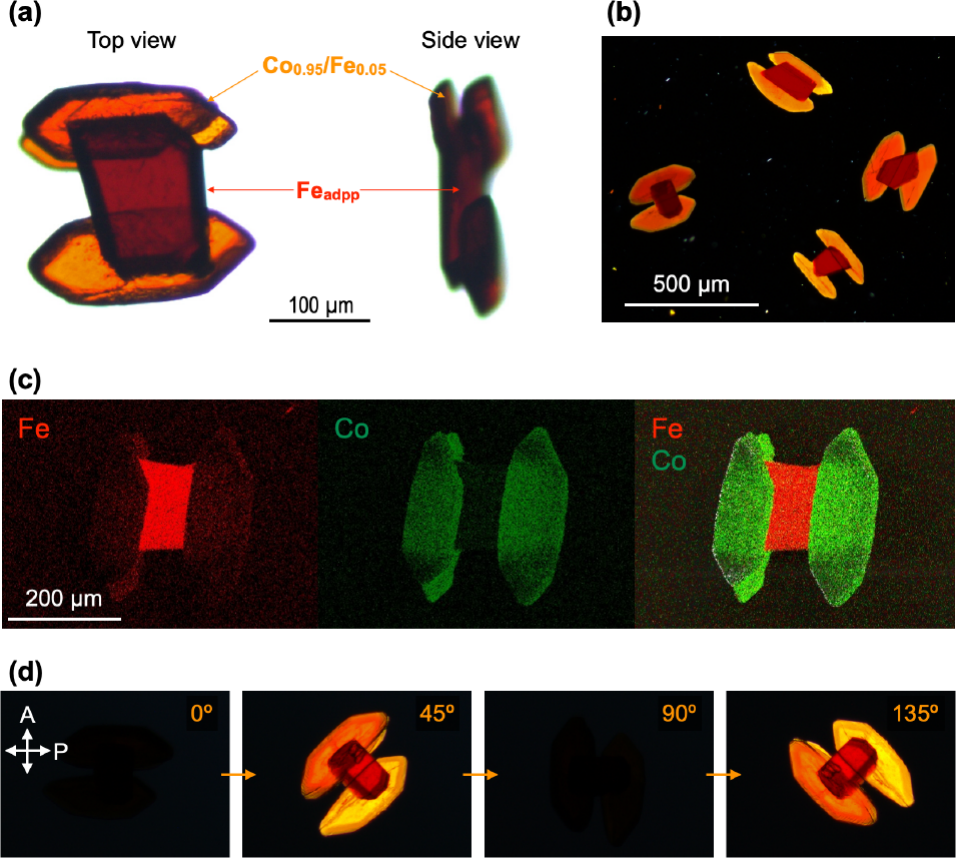
(a) Optical microscopy images of a **Fe**_**adpp**_ **@Co**_**0.95**_ **/Fe**_**0.05**_ dumbbell-like crystal, shown from top and side views. (b) POM image of **Fe**_**adpp**_ **@Co**_**0.95**_ **/Fe**_**0.05**_ dumbbell-like crystals. (c) SEM-EDX elemental mapping of a representative **Fe**_**adpp**_ **@Co**_**0.95**_ **/Fe**_**0.05**_ crystal, showing Fe (red), Co (green), and their overlay. (d) POM images of a **Fe**_**adpp**_ **@Co**_**0.95**_ **/Fe**_**0.05**_ dumbbell-like crystal taken at rotation angles of 0°, 45°, 90°, and 135° relative to the initial orientation. White arrows indicate the polarization directions of the polarizer (P) and analyzer (A). Orange numbers denote the rotation angles relative to the starting position.

Analysis of the crystallographic facets of the **Co**_**0.95**_ **/Fe**_**0.05**_ and **Fe**_**adpp**_ segments in **Fe**_**adpp**_ **@Co**_**0.95**_ **/Fe**_**0.05**_ further confirmed that secondary nucleation is completely suppressed on the *ac* plane, where the lattice mismatch is largest (Figure S11). This suppression of secondary nucleation on highly mismatched facets can be rationalized by increased interfacial free energy associated with lattice mismatch, which is consistent with the prolonged induction periods observed for shell growth at lower Fe contents (Figure 5i). When a sample of the dumbbell-like crystal was rotated in 45° steps under crossed nicols, the first and second segments displayed synchronized changes in birefringence, repeatedly producing bright and dark images at identical rotation angles (Figure 6d). Therefore, the molecular orientations in the two segments are the same. These results establish that facet-selective crystallization in this system is directed by anisotropic lattice mismatch at the heterointerface.

## Conclusions

We have demonstrated a strategy for constructing heterostructured molecular crystals composed of metal complexes by integrating solid-solution chemistry with on-seed-surface crystallization. As exemplified by the failure of the heterostructured crystal consisting of **Co**_**adpp**_ and **Fe**_**adpp**_, heterojunction between different molecular crystals is difficult to achieve, even when the constituent molecular structures are closely similar to each other. In the present strategy, solid-solution crystals are employed as secondary components that allow lattice matching between the seed and the secondary segment, thereby enabling on-seed-surface crystallization and the formation of **Fe**_**adpp**_ **@Co**_**1–** ***x***_ **/Fe**_***x***_ core–shell crystals. Crucially, the anisotropic lattice mismatch along crystallographic directions, which arises at the heterojunction by varying the solid-solution composition, governs facet-selective growth of secondary segments from a seed crystal. Consequently, dumbbell-like crystals can be obtained by tuning compositional ratio of the solid solution. Such dumbbell-like heterostructures with spatially confined heterointerfaces provide a platform for exploring how interfacial lattice mismatch influences spin and optoelectronic properties in molecular crystals.

While solid–solutions have long been used as a means to tune bulk properties through compositional variation, their relevance to on-seed-surface crystallization has remained largely unexplored. As demonstrated here, integrating solid–solution-mediated lattice matching with on-seed-surface crystallization can serve as a rational strategy for constructing heterostructured molecular crystals, which provide an opportunity to explore emergent functions that arise when multiple molecules are integrated in a single-crystalline form with distinct domains and interfaces.

## Supplementary Material



## Notes

Details of synthesis, single-crystal X-ray structures, optical and polarized optical microscopy data, XRF data, and SEM-EDX data (PDF)
