# Supplementary material for: Facet-Selective Growth of Heterostructured Molecular Crystals Enabled by a Solid-Solution-Mediated Strategy
Source: JACS Au. 2026 Mar 20;6(4):2473–9. doi: 10.1021/jacsau.6c00053 (PMC13126160; doi:10.1021/jacsau.6c00053)
Supplement: Supplementary file 1 [file au6c00053_si_001.pdf]

Supporting Information

Facet-Selective Growth of Heterostructured Molecular Crystals Enabled by a Solid-Solution-Mediated Strategy

Tomoya Fukui,*[a,b,c] Masahiro Tsuchiya,[a,b] Naoya Mita,[a,b] and Takanori Fukushima[a,b,c]

[a] Laboratory for Chemistry and Life Science, Institute of Integrated Research, Institute of Science Tokyo, 4259 Nagatsuta, Midori-ku, Yokohama 226-8501, Japan.

[b] Department of Chemical Science and Engineering, School of Materials and Chemical Technology, Institute of Science Tokyo, 4259 Nagatsuta, Midori-ku, Yokohama 226-8501, Japan.

[c] Research Center for Autonomous Systems Materialogy (ASMat), Institute of Integrated Research, Institute of Science Tokyo, 4259 Nagatsuta, Midori-ku, Yokohama 226-8501, Japan.

*To whom correspondence should be addressed

E-mail: fukui@cls.iir.isct.ac.jp (To.F.)

Table of Contents

1. Materials ········· S2
2. Methods ········· S2
3. Synthesis ········· S2
4. Single-Crystal X-ray Crystallography ········· S3
5. Supporting Figures ········· S5
6. Supporting Tables ········· S10
7. Supporting References ········· S12

1. Materials

Unless otherwise stated, all commercial reagents were used as received. 2,6-Bis(pyrazol-1-yl)pyridine-4-carboxamide (adpp) was synthesized according to the procedures reported previously.[S1]

2. Methods

Optical microscopy (OM) and polarized optical microscopy (POM) were performed on a Nikon model Eclipse LV100POL microscope. Real-time observation of crystallization processes was performed using a Plugable model USB2-MICRO-250X digital microscope, and images obtained were analyzed using ImageJ[S2] from the U.S. National Institutes of Health. Scanning electron microscopy (SEM) and energy dispersive X-ray spectroscopy (EDX) were performed using a JEOL model JSM-6610LA scanning electron microscope equipped with a JEOL model JED-2300 spectroscope with the relevant crystal samples immobilized on a carbon tape. X-ray fluorescence (XRF) spectroscopy was carried out on a BRUKER model S2-RANGER spectrometer. Powder X-ray diffraction (PXRD) measurements were performed on a RIGAKU model XtaLAB Synergy-DW diffractometer system equipped with a HyPix-6000 detector, using Cu-$K\alpha$ radiation (λ = 1.54184 Å) at 93 K under a cold nitrogen gas stream.

3. Synthesis

$[Fe(adpp)_2](BF_4)_2$ (Fe_{adpp}). $Fe(BF_4)_2{\bullet}6H_2O$ (2.3 mg, 6.8 μmol) was added to an acetonitrile suspension (0.8 mL) of adpp (4.3 mg, 17 μmol), and the resulting solution was stirred at 25 °C for 15 min and then filtered to remove any insoluble materials. A portion of the filtrate (0.20 mL) was slowly placed onto an acetonitrile solution of tetrabutylammonium tetrafluoroborate (6.6 M, 0.05 mL) prepared in advance in a microtube (diameter = 8 mm). After standing at 25 °C for two days, dark-red block-shaped crystals of **Fe_{adpp}** were collected by filtration (3.2 mg, 64% yield).

$[Co(adpp)_2](BF_4)_2{\bullet}(CH_3CN)_n{\bullet}(H_2O)_m$ (Co_{adpp}). $Co(BF_4)_2{\bullet}6H_2O$ (3.7 mg, 11 μmol) was added to an acetonitrile suspension (0.8 mL) of adpp (5.7 mg, 22 μmol), and the resulting solution was stirred at 25 °C for 15 min and then filtered to remove any insoluble materials. Diethyl ether vapor was allowed to diffuse into the filtrate at 25 °C. After standing for one day, orange rod-shaped crystals of **Co_{adpp}** were collected by filtration.

General procedure for the preparation of solid solutions. $Fe(BF_4)_2{\bullet}6H_2O$ (1.0, 2.0, 3.0, 4.0, or 5.0 μmol) and $Co(BF_4)_2{\bullet}6H_2O$ (9.0, 8.0, 7.0, 6.0, or 5.0 μmol, respectively) were added to an acetonitrile suspension (0.8 mL) of adpp (5.2 mg, 20 μmol), and the resulting solution was stirred at 25 °C for 15 min and then filtered to remove any insoluble materials. Diethyl ether vapor was allowed to diffuse into the filtrate at 25 °C. After standing for one day, solid-solution crystals of $[Co_{1-x}Fe_x(adpp)_2](BF_4)_2$ (**Co_{1-x}/Fe_x**; x denotes the feed ratio of Fe to Co) were collected by filtration.

General procedure for on-seed-surface crystallization. Freshly prepared **Fe_{adpp}** crystals (0.40 mg, 0.54 μmol) were added to an acetonitrile solution (0.4 mL) containing adpp (1.4 mg, 5.6 μmol), $Fe(BF_4)_2$•$6H_2O$ (0, 0.14, 0.28, 0.56, 0.84, 1.12, or 1.4 μmol) and $Co(BF_4)_2$•$6H_2O$ (2.80, 2.66, 2.52, 2.24, 1.96, 1.68, or 1.4 μmol, respectively) in a glass microtube (volume = 1.75 mL, diameter = 4.6 mm). Diethyl ether vapor was allowed to diffuse into the mixture at 25 °C under ambient air. In the absence of $Fe(BF_4)_2$•$6H_2O$, **Fe_{adpp}** and **Co_{adpp}** crystallized separately after standing for one day. In the presence of $Fe(BF_4)_2$•$6H_2O$, heterostructured crystals of **Fe_{adpp}@Co_{1-x}/Fe_x** (x = 0.05–0.5) formed after standing for one day and were collected by filtration.

4. Single-Crystal X-ray Crystallography

Single crystals were coated with immersion oil (type B: Code 1248, Cargille Laboratories, Inc.) and mounted on a MicroMount (MiTeGen, LLC). Diffraction data were collected at 93 K under a cold nitrogen gas stream on a RIGAKU model XtaLAB Synergy-DW diffractometer system equipped with a HyPix-6000 detector, using Cu $K\alpha$ radiation (λ = 1.54184 Å).

Crystal data for $C_{24}H_{20}N_{12}O_2FeB_2F_8$ (Fe_{adpp}): Dark-red blocks, 0.17 × 0.14 × 0.07 mm^3, monoclinic, $P2_1/n$, a = 9.24290(10) Å, b = 32.5079(2) Å, c = 10.30380(10) Å, β = 109.8590(10)°, V = 2911.84(5) $Å^3$, Z = 4, ρ_{calcd} = 1.683 g cm^{-3}, T = 93.15 K, μ = 5.072 mm^{-1}, 53162 reflections measured, 5828 unique reflections, 442 parameters, R_{int} = 0.0301, Completeness to theta = 74.657°: 97.6%, Absorption correction: multi-scan, GOF = 1.057, $R1$ = 0.0381 ($I > 2\sigma(I)$), w$R2$ = 0.0964 (all data), $\Delta\rho_{min,\ max}$ = –0.67, 0.89 e $Å^{-3}$, CCDC-2468322.

Crystal data for $C_{24}H_{20}N_{12}O_2CoB_2F_8$ (Co_{adpp}): Yellow blocks, 0.15 × 0.10 × 0.06 mm^3, monoclinic, $P2/c$, a = 26.7555(5) Å, b = 8.04350(10) Å, c = 34.6622(7) Å, β = 106.640(2)°, V = 7147.2(2) $Å^3$, Z = 8, ρ_{calcd} = 1.377 g cm^{-3}, T = 93 K, μ = 4.513 mm^{-1}, 14696 reflections measured, 14696 unique reflections, 956 parameters, Completeness to theta = 76.878°: 97.5%, Absorption correction: multi-scan, GOF = 1.827, $R1$ = 0.1414 ($I > 2\sigma(I)$), w$R2$ = 0.4128 (all data), $\Delta\rho_{min,\ max}$ = –0.55, 1.45 e $Å^{-3}$, CCDC-2468323.

Crystal data for $C_{24}H_{20}N_{12}O_2Fe_{0.5}Co_{0.5}B_2F_8$ ($Co_{0.5}$/$Fe_{0.5}$): Dark-orange plates, 0.33 × 0.14 × 0.08 mm^3, monoclinic, $P2_1/n$, a = 9.37180(10) Å, b = 32.8070(3) Å, c = 10.15980(10) Å, β = 109.310(1)°, V = 2948.01(5) $Å^3$, Z = 4, ρ_{calcd} = 1.666 g cm^{-3}, T = 93.15 K, μ = 5.240 mm^{-1}, 57827 reflections measured, 6077 unique reflections, 437 parameters, R_{int} = 0.0348, Completeness to theta = 76.606°: 98.0%, Absorption correction: multi-scan, GOF = 1.068, $R1$ = 0.0449 ($I > 2\sigma(I)$), w$R2$ = 0.1076 (all data), $\Delta\rho_{min,\ max}$ = –1.45, 0.47 e $Å^{-3}$, CCDC-2468324.

Crystal data for $C_{24}H_{20}N_{12}O_2Fe_{0.5}Co_{0.5}B_2F_8$ ($Co_{0.5}$/$Fe_{0.5}$ in the shell of **Fe_{adpp}@$Co_{0.5}$/$Fe_{0.5}$):** Dark-orange plates, 0.61 × 0.19 × 0.13 mm^3, monoclinic, $P2_1/n$, a = 9.3485(2) Å, b = 32.7471(5) Å, c = 10.1913(2) Å, β = 109.452(2)°, V = 2941.84(10) $Å^3$, Z = 4, ρ_{calcd} = 1.670 g cm^{-3}, T = 93.15 K, μ = 5.251 mm^{-1}, 51918 reflections measured, 5944 unique reflections, 437 parameters, R_{int} =

0.0713, Completeness to theta = 74.967°: 98.1%, Absorption correction: multi-scan, GOF = 1.086, $R1 = 0.0713$ ($I > 2\sigma(I)$), $wR2 = 0.1651$ (all data), $\Delta\rho_{min, max} = -1.61, 0.85$ e Å^{-3}, CCDC-2468327.

Crystal data for $C_{24}H_{20}N_{12}O_2Fe_{0.4}Co_{0.6}B_2F_8$ ($Co_{0.6}/Fe_{0.4}$ in the shell of **Fe_{adpp}@$Co_{0.6}/Fe_{0.4}$):** Dark-orange plates, 0.36 × 0.13 × 0.06 mm^3, monoclinic, $P2_1/n$, $a = 9.34430(10)$ Å, $b = 32.7130(4)$ Å, $c = 10.20540(10)$ Å, $\beta = 109.435(2)$°, $V = 2941.83(7)$ Å^3, $Z = 4$, $\rho_{calcd} = 1.670$ g cm^{-3}, $T = 93.15$ K, $\mu = 5.298$ mm^{-1}, 53245 reflections measured, 5684 unique reflections, 442 parameters, $R_{int} = 0.0419$, Completeness to theta = 71.401°: 99.1%, Absorption correction: multi-scan, GOF = 1.064, $R1 = 0.0376$ ($I > 2\sigma(I)$), $wR2 = 0.0972$ (all data), $\Delta\rho_{min, max} = -0.44, 0.68$ e Å^{-3}, CCDC-2468328.

Crystal data for $C_{24}H_{20}N_{12}O_2Fe_{0.3}Co_{0.7}B_2F_8$ ($Co_{0.7}/Fe_{0.3}$ in the shell of **Fe_{adpp}@$Co_{0.7}/Fe_{0.3}$):** Dark-orange plates, 0.36 × 0.13 × 0.11 mm^3, monoclinic, $P2_1/n$, $a = 9.3845(3)$ Å, $b = 32.8215(9)$ Å, $c = 10.1473(4)$ Å, $\beta = 109.311(4)$°, $V = 2949.65(18)$ Å^3, $Z = 4$, $\rho_{calcd} = 1.667$ g cm^{-3}, $T = 93.15$ K, $\mu = 5.330$ mm^{-1}, 19612 reflections measured, 5879 unique reflections, 442 parameters, $R_{int} = 0.0519$, Completeness to theta = 76.431°: 94.9%, Absorption correction: multi-scan, GOF = 1.073, $R1 = 0.0651$ ($I > 2\sigma(I)$), $wR2 = 0.1434$ (all data), $\Delta\rho_{min, max} = -0.71, 0.53$ e Å^{-3}, CCDC-2468325.

Crystal data for $C_{24}H_{20}N_{12}O_2Fe_{0.2}Co_{0.8}B_2F_8$ ($Co_{0.8}/Fe_{0.2}$ in the shell of **Fe_{adpp}@$Co_{0.8}/Fe_{0.2}$):** Orange plates, 0.2 × 0.13 × 0.07 mm^3, monoclinic, $P2_1/n$, $a = 9.41620(10)$ Å, $b = 32.8642(5)$ Å, $c = 10.13190(10)$ Å, $\beta = 109.2520(10)$°, $V = 2960.04(6)$ Å^3, $Z = 4$, $\rho_{calcd} = 1.662$ g cm^{-3}, $T = 93.15$ K, $\mu = 5.357$ mm^{-1}, 63402 reflections measured, 6085 unique reflections, 442 parameters, $R_{int} = 0.0426$, Completeness to theta = 76.597°: 97.7%, Absorption correction: multi-scan, GOF = 1.097, $R1 = 0.0471$ ($I > 2\sigma(I)$), $wR2 = 0.1139$ (all data), $\Delta\rho_{min, max} = -0.61, 0.52$ e Å^{-3}, CCDC-2468330.

Crystal data for $C_{24}H_{20}N_{12}O_2Fe_{0.1}Co_{0.9}B_2F_8$ ($Co_{0.9}/Fe_{0.1}$ in the shell of **Fe_{adpp}@$Co_{0.9}/Fe_{0.1}$):** Orange plates, 0.21 × 0.10 × 0.09 mm^3, monoclinic, $P2_1/n$, $a = 9.49470(10)$ Å, $b = 33.0037(3)$ Å, $c = 10.04510(10)$ Å, $\beta = 109.0790(10)$°, $V = 2974.82(5)$ Å^3, $Z = 4$, $\rho_{calcd} = 1.654$ g cm^{-3}, $T = 93$ K, $\mu = 5.376$ mm^{-1}, 56007 reflections measured, 6171 unique reflections, 442 parameters, $R_{int} = 0.0657$, Completeness to theta = 76.706°: 98.6%, Absorption correction: multi-scan, GOF = 1.054, $R1 = 0.0502$ ($I > 2\sigma(I)$), $wR2 = 0.1418$ (all data), $\Delta\rho_{min, max} = -0.40, 0.61$ e Å^{-3}, CCDC-2468326.

Crystal data for $C_{24}H_{20}N_{12}O_2Fe_{0.05}Co_{0.95}B_2F_8$ ($Co_{0.95}/Fe_{0.05}$ in the shell of **Fe_{adpp}@$Co_{0.95}/Fe_{0.05}$):** Orange blocks, 0.30 × 0.12 × 0.08 mm^3, monoclinic, $P2_1/n$, $a = 9.55580(10)$ Å, $b = 33.0814(4)$ Å, $c = 9.9838(2)$ Å, $\beta = 109.046(2)$°, $V = 2983.30(8)$ Å^3, $Z = 4$, $\rho_{calcd} = 1.650$ g cm^{-3}, $T = 93.15$ K, $\mu = 5.384$ mm^{-1}, 53563 reflections measured, 5760 unique reflections, 442 parameters, $R_{int} = 0.0503$, Completeness to theta = 71.236°: 99.3%, Absorption correction: multi-scan, GOF = 1.107, $R1 = 0.0476$ ($I > 2\sigma(I)$), $wR2 = 0.1151$ (all data), $\Delta\rho_{min, max} = -0.37, 0.61$ e Å^{-3}, CCDC-2468329.

5. Supporting Figures

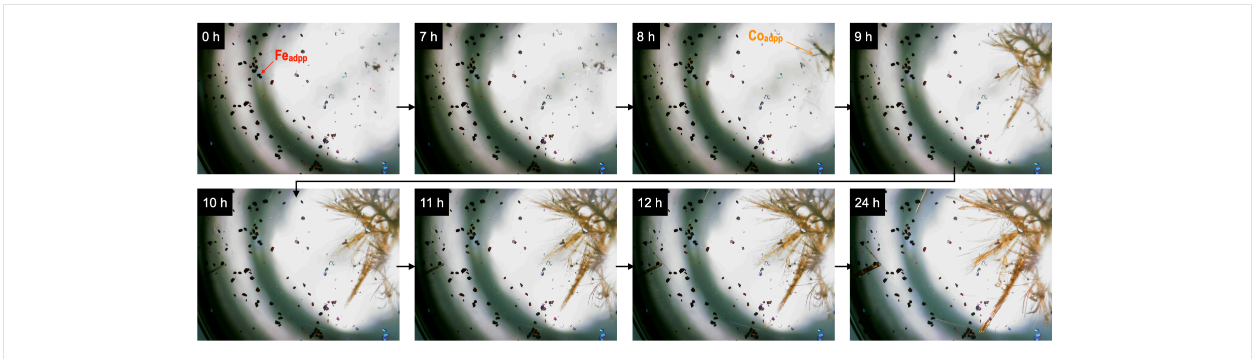

Figure S1. OM images of the growth process of **Co_{adpp}** at different time points. No on-seed-surface growth of **Co_{adpp}** from **Fe_{adpp}** seeds was observed.

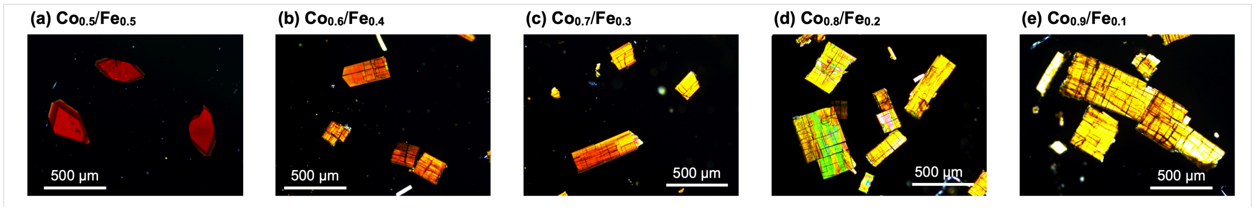

Figure S2. POM images of solid-solution crystals of (a) **$Co_{0.5}/Fe_{0.5}$**, (b) **$Co_{0.6}/Fe_{0.4}$**, (c) **$Co_{0.7}/Fe_{0.3}$**, (d) **$Co_{0.8}/Fe_{0.2}$**, and (e) **$Co_{0.9}/Fe_{0.1}$**.

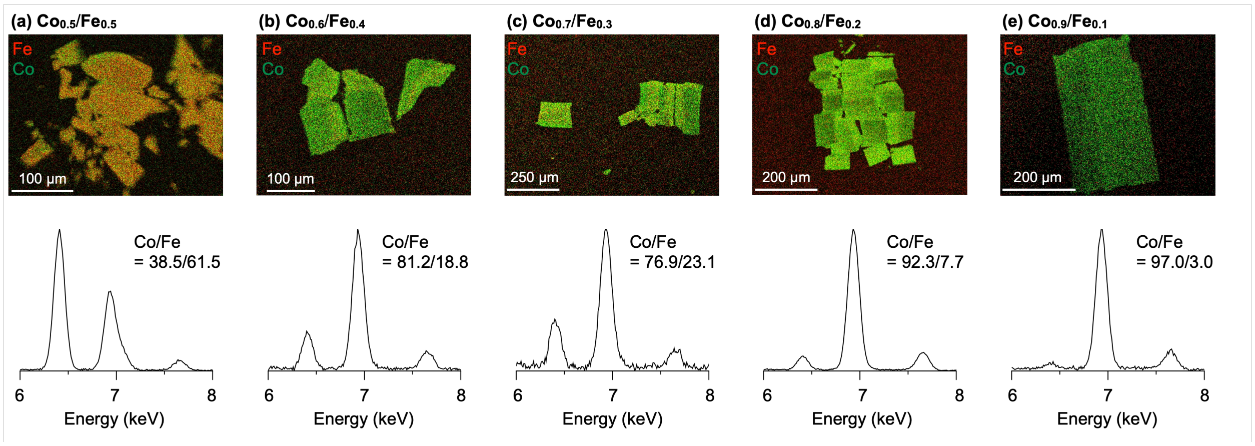

Figure S3. SEM-EDX elemental mapping images (top) and the corresponding EDX spectra (bottom) of solid-solution crystals of (a) **$Co_{0.5}/Fe_{0.5}$**, (b) **$Co_{0.6}/Fe_{0.4}$**, (c) **$Co_{0.7}/Fe_{0.3}$**, (d) **$Co_{0.8}/Fe_{0.2}$**, and (e) **$Co_{0.9}/Fe_{0.1}$**.

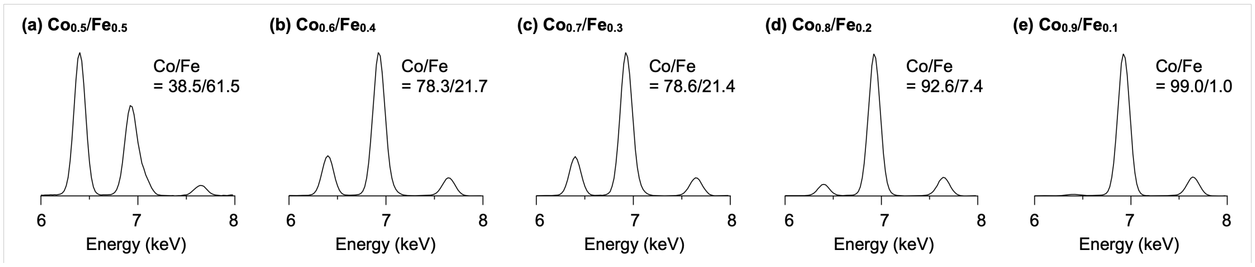

Figure S4. XRF spectra of solid-solution crystals of (a) **$Co_{0.5}/Fe_{0.5}$**, (b) **$Co_{0.6}/Fe_{0.4}$**, (c) **$Co_{0.7}/Fe_{0.3}$**, (d) **$Co_{0.8}/Fe_{0.2}$**, and (e) **$Co_{0.9}/Fe_{0.1}$**.

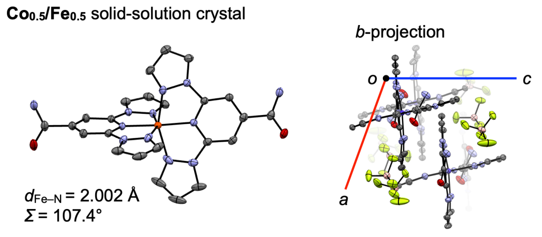

Figure S5. X-ray crystal structure (molecular and packing views) of **$Co_{0.5}/Fe_{0.5}$** at 93 K with atomic displacement parameters set at 50% probability. Color code: carbon = gray, nitrogen = blue, oxygen = red, cobalt/iron = orange, fluorine = green, boron = pink. Hydrogen atoms have been omitted for clarity.

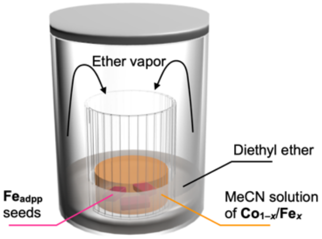

Figure S6. Schematic illustration of the experimental setup for on-seed-surface crystallization of **Co_{1-x}/Fe_x** using **Fe_{adpp}** seeds.

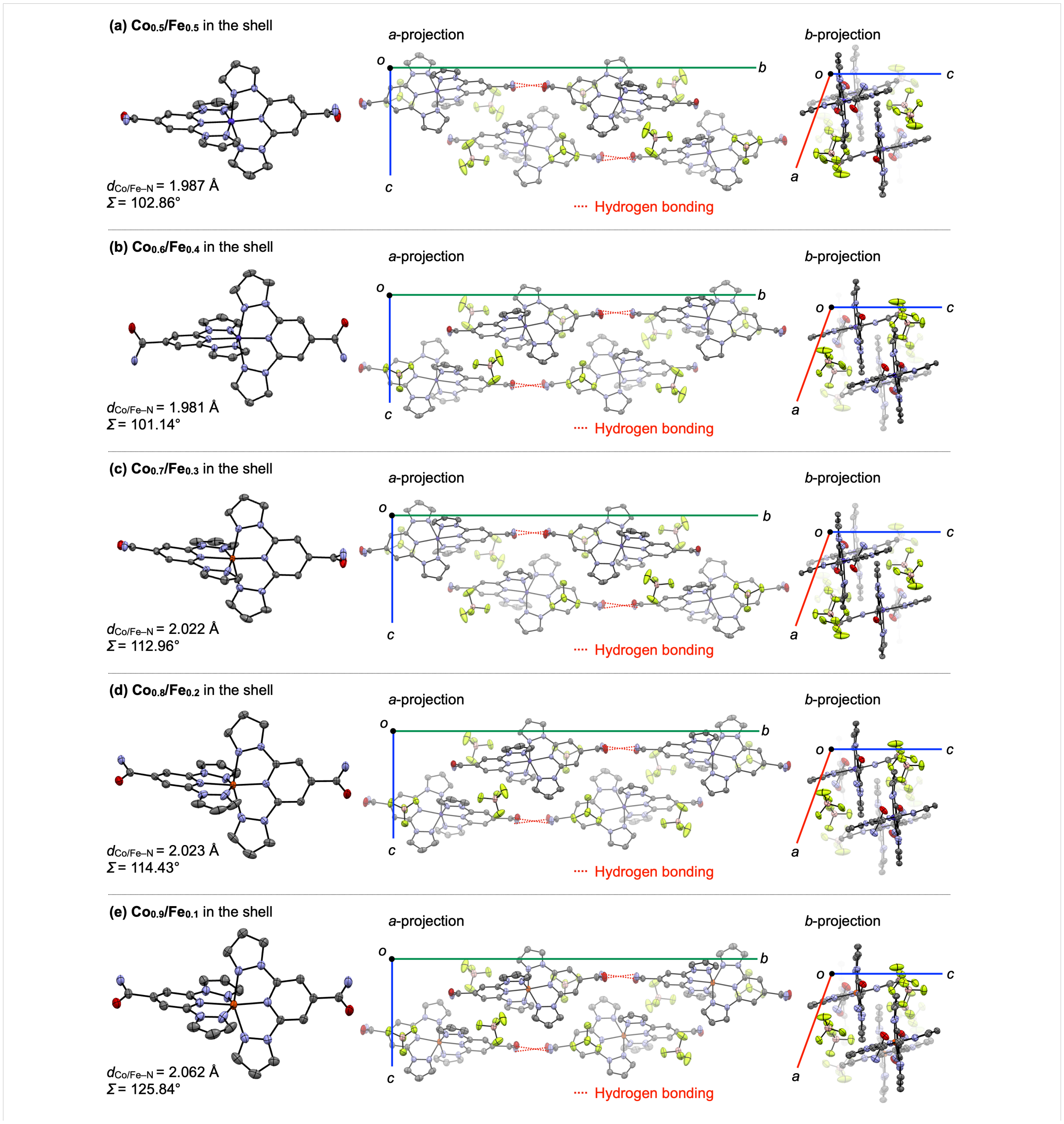

Figure S7. X-ray crystal structures (molecular and packing views) of (a) **$Co_{0.5}/Fe_{0.5}$**, (b) **$Co_{0.6}/Fe_{0.4}$**, (c) **$Co_{0.7}/Fe_{0.3}$**, (d) **$Co_{0.8}/Fe_{0.2}$**, and (e) **$Co_{0.9}/Fe_{0.1}$** shells at 93 K with atomic displacement parameters set at 50% probability. Color code: carbon = gray, nitrogen = blue, oxygen = red, cobalt/iron = orange or purple, fluorine = green, boron = pink. Hydrogen atoms have been omitted for clarity.

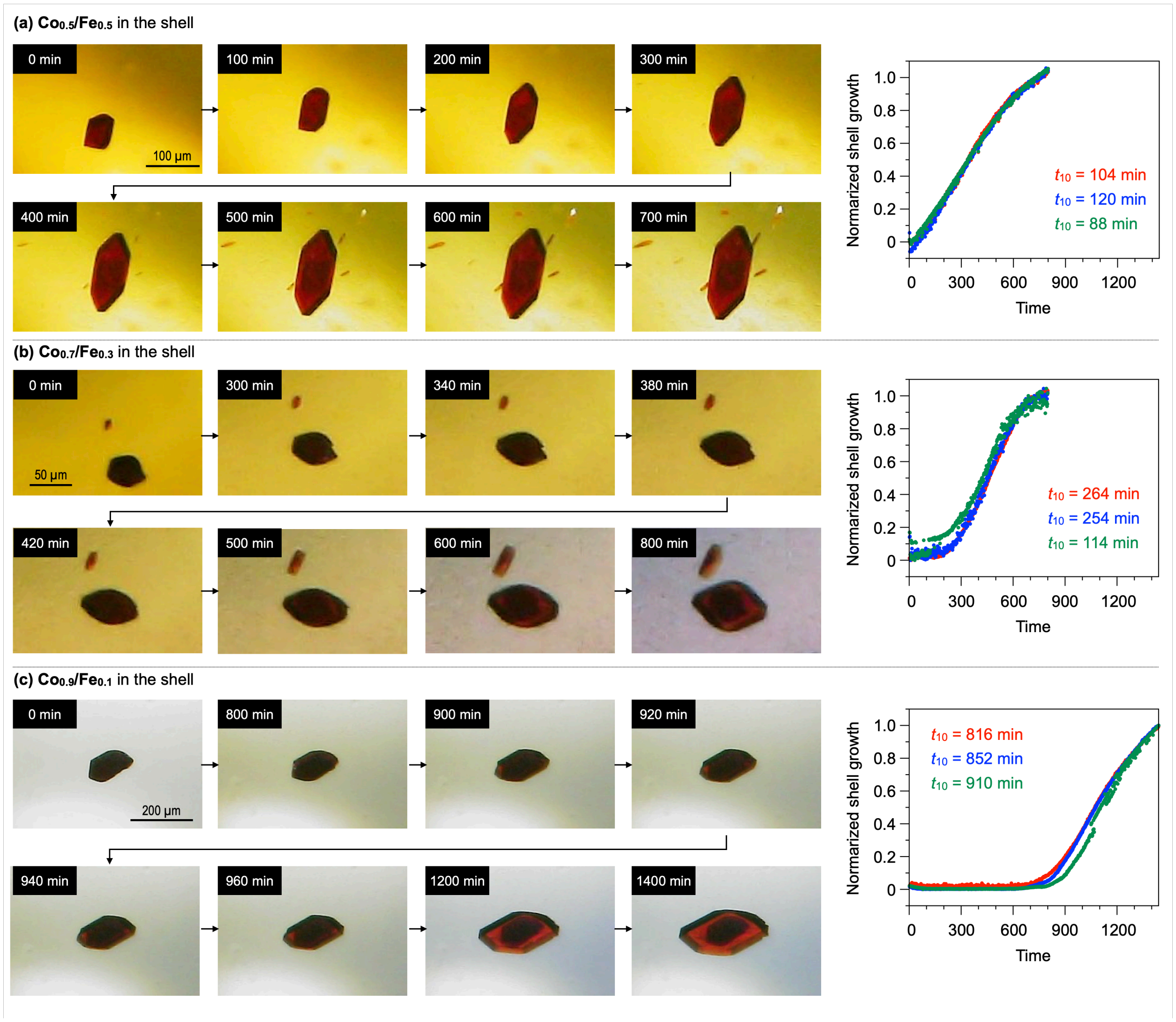

Figure S8. OM images at different time points (left) and plots of normalized shell area as a function of time (right) for on-seed-surface crystallization of (a) **Fe$_{adpp}$@Co$_{0.5}$/Fe$_{0.5}$**, (b) **Fe$_{adpp}$@Co$_{0.7}$/Fe$_{0.3}$**, and (c) **Fe$_{adpp}$@Co$_{0.9}$/Fe$_{0.1}$**. The shell area was normalized to its final value for each crystal. The induction period (t_{10}) was defined as the time required for the shell area to reach 10% of its final value.

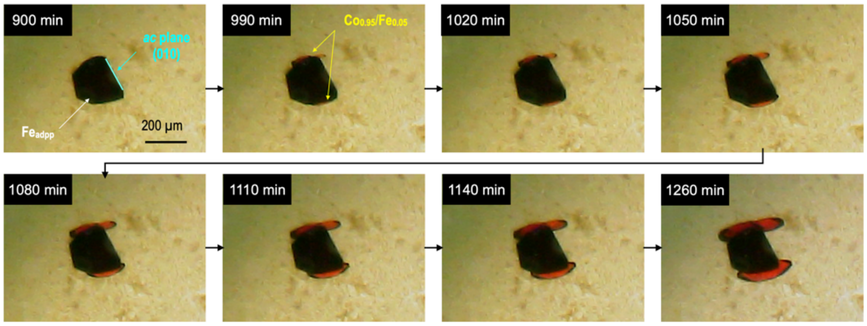

Figure S9. OM images of the growth process of a **Fe$_{adpp}$@Co$_{0.95}$/Fe$_{0.05}$** dumbbell-like crystal at different time points.

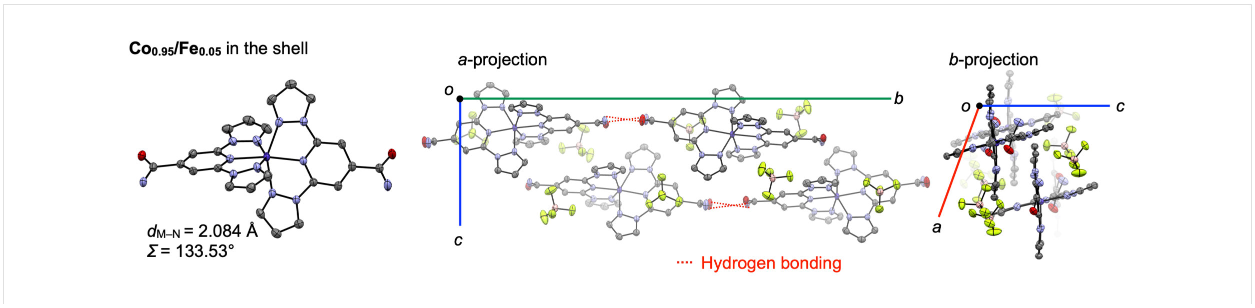

Figure S10. X-ray crystal structure (molecular and packing views) of **Co$_{0.95}$/Fe$_{0.05}$**, obtained by truncation from a **Fe$_{adpp}$@Co$_{0.95}$/Fe$_{0.05}$** crystal, measured at 93 K with atomic displacement parameters set at 50% probability. Color code: carbon = gray, nitrogen = blue, oxygen = red, cobalt/iron = purple, fluorine = green, boron = pink. Hydrogen atoms have been omitted for clarity.

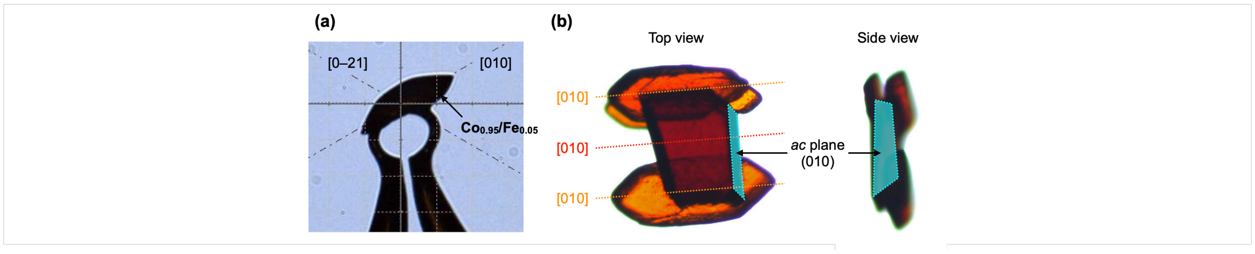

Figure S11. (a) A photograph of the **Co$_{0.95}$/Fe$_{0.05}$** segment of a **Fe$_{adpp}$@Co$_{0.95}$/Fe$_{0.05}$** crystal on a MicroMount. The dashed lines represent the reciprocal lattice vectors determined from single-crystal X-ray analysis, labeled as [*hkl*]. (b) Top and side views of a **Fe$_{adpp}$@Co$_{0.95}$/Fe$_{0.05}$** dumbbell-like crystal, with crystallographic axes and the (010) plane identified based on the orientation analysis in (a).

6. Supporting Tables

Table S1. Average M–N bond lengths ($d_{M–N}$) (M = Fe, Co, and Co/Fe) and octahedral distortion parameters (Σ) for $\mathbf{M_{adpp}}$ (M = Fe and Co) and $\mathbf{Co_{0.5}/Fe_{0.5}}$.

	$\mathbf{Fe_{adpp}}$	$\mathbf{Co_{adpp}}$		$\mathbf{Co_{0.5}/Fe_{0.5}}$
$d_{M–N}$ (Å)	1.937	2.117	2.107	2.002
Σ (°)	88.51	141.81	131.76	107.4

Table S2. Elemental ratios in the shell region of $\mathbf{Fe_{adpp}@Co_{1-x}/Fe_x}$ (x = 0.1–0.5) determined by SEM-EDX analysis (Figure 5).

Content	$\mathbf{Co_{0.5}/Fe_{0.5}}$ shell	$\mathbf{Co_{0.6}/Fe_{0.4}}$ shell	$\mathbf{Co_{0.7}/Fe_{0.3}}$ shell	$\mathbf{Co_{0.8}/Fe_{0.2}}$ shell	$\mathbf{Co_{0.9}/Fe_{0.1}}$ shell
Fe (%)	48.81	35.89	26.88	23.28	10.96
Co (%)	51.19	64.11	73.12	76.72	89.04

Table S3. Lattice constant mismatches between the $\mathbf{Fe_{adpp}}$ core and the $\mathbf{Co_{1-\mathit{x}}/Fe_{\mathit{x}}}$ shells (x = 0.05–0.5).

	$\mathbf{Co_{0.5}/Fe_{0.5}}$ shell	$\mathbf{Co_{0.6}/Fe_{0.4}}$ shell	$\mathbf{Co_{0.7}/Fe_{0.3}}$ shell	$\mathbf{Co_{0.8}/Fe_{0.2}}$ shell	$\mathbf{Co_{0.9}/Fe_{0.1}}$ shell	$\mathbf{Co_{0.95}/Fe_{0.05}}$ shell
Percentage difference in *a* (%)	1.14	1.10	1.53	1.87	2.72	3.39
Percentage difference in *b* (%)	0.73	0.63	0.96	1.10	1.53	1.76
Percentage difference in *c* (%)	1.09	0.96	1.52	1.67	2.51	3.11
Percentage difference in *β* (%)	0.37	0.39	0.50	0.55	0.71	0.74

Percentage differences were calculated using the following equation: 100 × (j–k)/k, where j is the lattice constant (a, b, c, or β) of the $\mathbf{Co_{1-\mathit{x}}/Fe_{\mathit{x}}}$ (x = 0.05–0.5) shells, and k is the corresponding lattice constant of $\mathbf{Fe_{adpp}}$.

In Figure 5h, a pronounced and simultaneous decrease in lattice mismatch between x = 0.5 and 0.4 is observed. Its reproducibility was confirmed by independent single-crystal X-ray analyses. SEM-EDX measurements showed that the Fe/Co composition of the shells accurately reflects the corresponding metal feed ratios, excluding compositional inhomogeneity as the origin of this behavior. A closer inspection of the crystallographic parameters reveals that the β angle increases monotonically with decreasing Fe content, whereas the a, b, and c lattice constants deviate from a simple monotonic trend. Partial substitution between Co^{II} and Fe^{II} ions, which differ slightly in ionic radius and coordination geometry, may induce subtle changes in molecular packing, leading to a non-linear lattice response.

7. Supporting References

S1. Attwood, M.; Akutsu, H.; Martin, L.; Blundell, T. J.; Maguere, P. L.; Turner, S. S. Exceptionally high temperature spin crossover in amide-functionalised 2,6-bis(pyrazol-1-yl)pyridine iron(II) complex revealed by variable temperature raman spectroscopy and single crystal X-ray diffraction. *Dalton Trans.* **2021**, *50*, 11843–11851.

S2. Schneider, C. A.; Rasband, W. S.; Eliceiri, K. W. NIH Image to ImageJ: 25 years of image analysis. *Nat. Methods* **2012**, *9*, 671–675.
